# Epidemiology of gastrointestinal infections: lessons learned from syndromic testing, Region Zealand, Denmark

**DOI:** 10.1007/s10096-023-04642-5

**Published:** 2023-07-19

**Authors:** Rikke Lykke Johansen, Christian Højte Schouw, Tina Vasehus Madsen, Xiaohui Chen Nielsen, Jørgen Engberg

**Affiliations:** The Regional Department of Clinical Microbiology, Zealand University Hospital, Koege, Ingemannsvej 46, DK-4200 Slagelse, Denmark

**Keywords:** Gastrointestinal infections, Diarrhea, Syndromic testing, QIAstat-Dx, Multiplex PCR, Evaluation, Experience

## Abstract

The aim of this study was to investigate the value of syndromic diagnostic testing for a better understanding of the epidemiology of gastrointestinal infections in Denmark. Here we evaluated the QIAstat-Dx® Gastrointestinal (GI) Panel 1 assay on 18,610 fecal samples requested for analysis for enteric pathogens in Region Zealand, Denmark, in 1 year (October 1, 2021, to September 30, 2022). In total, 6905 (37%) samples were detected positive for one or more diarrhoeal bacteria, viruses, and protozoa. The most common bacterial, viral, and parasitic pathogens detected with the QIAstat-Dx® Gastrointestinal Panel 1 were EPEC (in patients ≥ 2 years of age) (*n* = 1420 (20.6%)), rotavirus (*n* = 948 (13.7%)), and *Cryptosporidium* spp. (*n* = 196 (2.84%)). We identified a large diversity in infections likely reflecting substantial differences in the epidemiology including origin of infections, mode of transmission, seasonality, age-dependent susceptibility to disease, severity, and travel history. All pathogens were detected as both single and coinfections. Viral infections peaked in March with a positive rate of 31.6%, and bacterial infections peaked in August with a positive rate of 35.3%. ETEC, *Shigella*/EIEC, EAEC, and *P. shigelloides* were most related to travel activity, and coinfections were frequent. The distribution of *C*_*t*_ values varied significantly between the pathogens, with the lowest *C*_*t*_ values (median 17–18) observed in astrovirus, adenovirus, and rotavirus. Our results highlight the value of providing extensive diagnostic testing on fecal samples for sufficient detection of relevant diarrhoeal pathogens for optimal clinical care.

## Introduction

Infectious gastroenteritis is one of the most common diseases throughout the world. It poses a global health problem, especially severe infections in infants, elderly, and immunocompromised patients can lead to hospitalization because of the increased risk of dehydration [1, 2]. Each year approximately 2 billion cases of acute diarrhea have been reported by The World Health Organization (WHO) and United Nations International Children’s Emergency Fund (UNICEF), including 1.9 million children under 5 years that are dying from acute diarrhea annually [1, 2]. A wide range of enteric pathogens, including bacteria, viruses, and protozoa, can cause infectious gastroenteritis. The pattern of clinical symptoms is seldom sufficient in identifying the aetiological agent. Therefore, rapid and accurate diagnosis is essential for appropriate patient treatment and infection control precautions [3].

Conventional methods for diarrhoeagenic bacteria involving stool culture, biochemical assays, and serologic assays can be time-consuming and have a lack of sensitivity. Multiplex polymerase chain reaction (PCR)-based assays have been a faster and more sensitive alternative to conventional methods. However, the number of pathogens included is limited for a single multiplex PCR. In recent years, commercial multiplex PCR panel testing systems, also known as syndromic panels, have been developed and implemented in microbiology laboratories [4]. Syndromic panels are designed for infectious pathogen diagnostics in patients with similar symptoms or syndrome and are characterized by short turn-around times, minimal hands-on time, and an automated workflow [4]. The implementation of the syndromic panel testing allows to extend the range of detectable pathogens independently of the clinical suspicion and to detect coinfections [5].

One of these platforms is the CE-IVD-marked QIAstat-Dx® with the Gastrointestinal Panel 1 (cat. no. 691411) (QIAGEN, Hilden, Germany) that can detect 24 gastrointestinal pathogens: 14 bacteria, 6 viruses, and 4 protozoa in a single run [6, 7].

Compared with other syndromic panel systems like BioFire FilmArray Gastrointestinal Panel (bioMérieux, Marcy-l’Étoile, France) and xTAG Gastrointestinal Pathogen Panel (Luminex, Austin, Texas, USA), the QIAstat-Dx® Gastrointestinal Panel has the advantage of generating and presenting cycle threshold (*C*_*t*_) values for the detected pathogen(s). The clinical utility of *C*_*t*_ values remains unclear for gastrointestinal infections; however, *C*_*t*_ values could provide a quantitative indication and might be a guide for clinical and infection control decisions [8].

We have verified the analytical spectrum of the QIAstat-Dx® Gastrointestinal Panel [7]. In this study, we evaluate the 1-year experience with the QIAstat-Dx® Gastrointestinal Panel as a routine diagnostic method for diarrheal infections in The Regional Department of Clinical Microbiology at Zealand University Hospital, Region Zealand, Denmark. We describe findings related to detected pathogens, including coinfections, age, seasonal variation, travel history, and characteristics of *C*_*t*_ values.

## Materials and methods

### Population

The Regional Department of Clinical Microbiology at Zealand University Hospital, Region Zealand, Denmark, provides diagnostic microbiological services for both in- and outpatients from a referring area covering approximately 800,000 inhabitants. History of travel was obtained from the electronic laboratory test request form, where travel information is mandatory.

### Fecal samples

All fecal samples submitted to the laboratory for analysis of gastrointestinal pathogens from October 1, 2021, to September 30, 2022, were included. The samples originated from both in- and outpatients and were actively collected by the healthcare service from hospitals and general practitioner (GP) clinics. On arrival in the laboratory, the samples were kept at ambient temperature until analysis, which was usually performed within 4 to 5 h of receipt. Analysis was performed 7 days a week, and the majority of samples were tested within 24 h of collection. If the laboratory received more than one fecal sample from the same patient within 7 days, only the first sample was analyzed.

### Analytical spectrum

The QIAstat-Dx® Gastrointestinal Panel detects the following pathogens: *Campylobacter* spp. (*C. jejuni*, *C. upsaliensis*, and *C. coli*), *Clostridioides difficile tcdA/tcdB*, enteroaggregative *E. coli* (EAEC), enteropathogenic *E. coli* (EPEC), enterotoxigenic *E. coli* (ETEC) *eltA*/*estA*, Shiga toxin–producing *E. coli* (STEC) *stx1/stx2*, Shiga toxin–producing *E. coli* (STEC) *stx1/stx2* O157, enteroinvasive *E. coli* (EIEC)/*Shigella* (*S. sonnei*, *S. flexneri*, *S. boydii*, and *S. dysenteriae*), *Plesiomonas shigelloides*, *Salmonella* spp., *Vibrio cholerae*, *Vibrio parahaemolyticus*, *Vibrio vulnificus*, *Yersinia enterocolitica*, *Cyclospora cayetanensis*, *Cryptosporidium* spp. (*C. parvum*, *C. hominis*, *C. felis*, and *C. meleagridis*), *Entamoeba histolytica*, *Giardia lamblia*, adenovirus F40/F41, norovirus GI and GII, rotavirus, and sapovirus (I, II, IV, and V).

### PCR analysis

The analysis with QIAstat-Dx® Gastrointestinal Panel 1 was carried out according to the manufacturer’s instructions and as previously described [7]. The analysis requires approximately 50–200 mg of feces collected with a flocked swab from the FaecalSwab sample collection system (Copan, Brescia, Italy) that has to be resuspended in 2 mL of Cary-Blair transport medium. A total of 200 μL of the FaecalSwab suspension was collected using a transfer pipette and loaded into the liquid sample port of a QIAstat-Dx® Gastrointestinal Panel cartridge. All reactions are performed by the closed QIAstat-Dx® system within the loaded cartridge and included lysis, extraction, amplification, and measurements of fluorescence of the amplified PCR products. The QIAstat-Dx® Analyzer Software interprets the results and generates test reports used to evaluate potential gastrointestinal pathogen findings. An internal control is included in the assay to monitor the quality of the reactions for a given sample. If the internal control is reported positive, all results are valid. If the internal control is reported negative, only positive results for targets are valid while negative results for targets are invalid. Running a sample with the QIAstat-Dx® Gastrointestinal Panel takes approximately 70 min/sample.

### Statistical analysis

The descriptive epidemiology data was analyzed with the software R version 4.2.1 (R Foundation for Statistical Computing, Vienna, Austria, https://www.r-project.org).

## Results

**Fig. 1:**
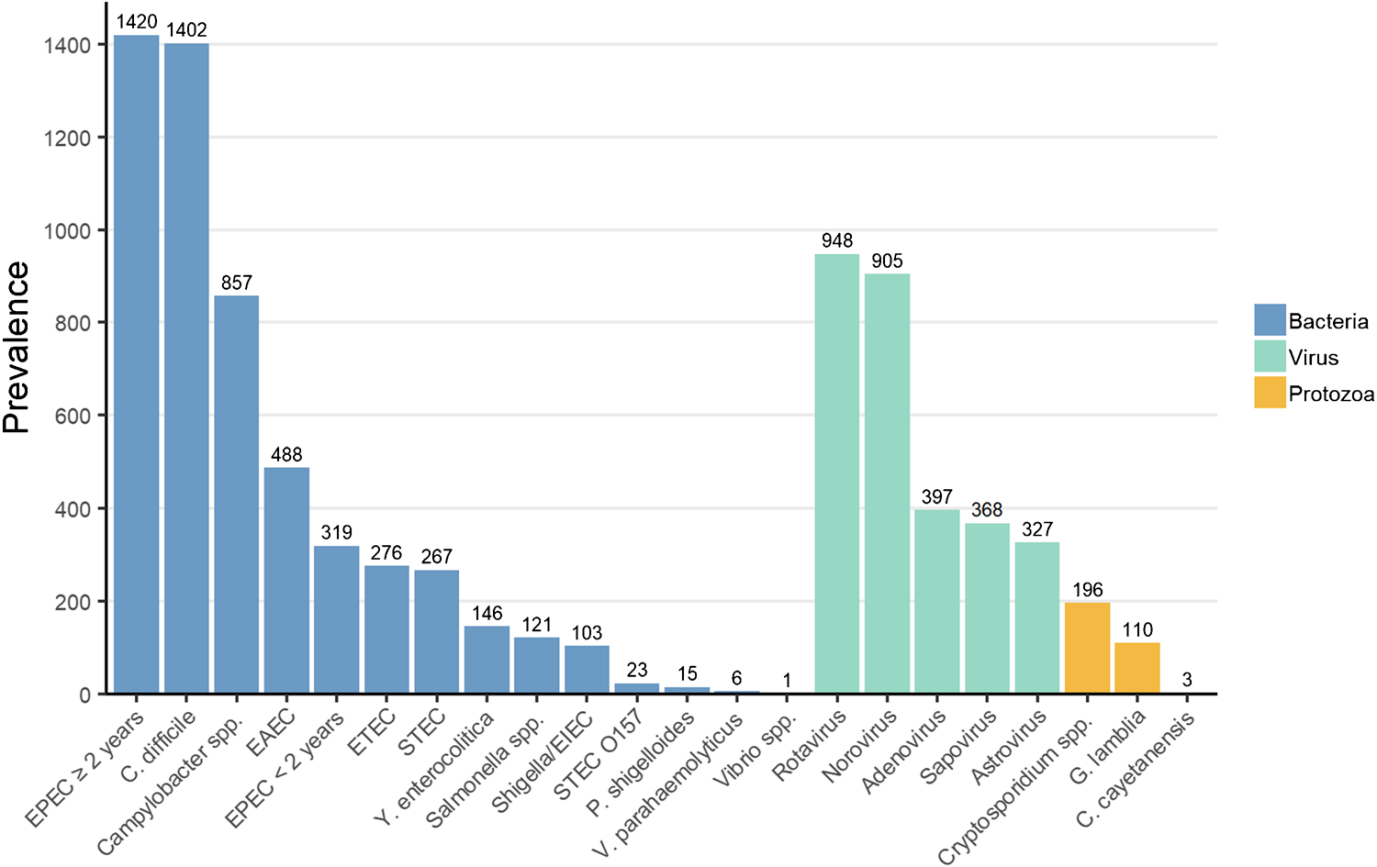
Positive fecal samples by the enteric pathogen, October 2021 to October 2022

A total of 18,610 fecal samples were included in this 1-year study from October 1, 2021, to September 30, 2022. The samples were from 10,211 females and 8399 males, with a median age of 54 years (range 0–103 years). A total of 6905 (37.1%) fecal samples were positive for one or more gastrointestinal pathogens. Bacterial pathogens were detected in 5444 (78.8%) samples and virus and protozoa in 2945 (42.7%) and 309 (4.5%) samples, respectively (Fig. 1). The most prevalent gastrointestinal pathogens were EPEC in patients ≥ 2 years of age (*n* = 1420 (20.6%)), *C. difficile* (*n* = 1402 (20.3%)), rotavirus (*n* = 948 (13.7%)), norovirus (*n* = 905 (13.1%)). and *Campylobacter* spp. (*n* = 857 (12.4%)), respectively. The prevalence of gastrointestinal infections and type of gastrointestinal pathogens were influenced by several factors, including patient age, travel, origin (in- or outpatients), and season.

**Fig. 2:**
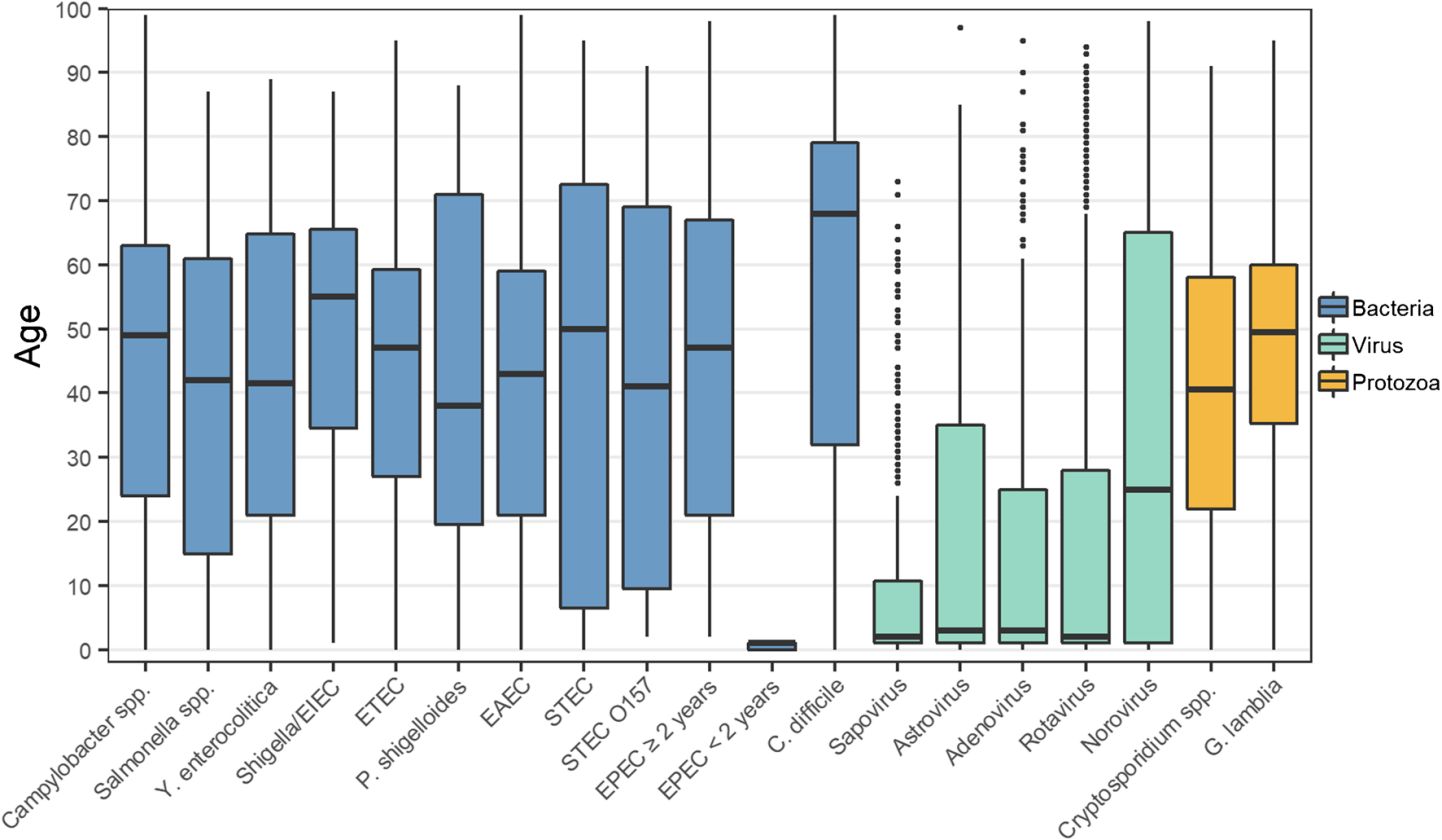
The median and quartiles of age by an enteric pathogen. Pathogens detected < 10 times are not presented

In general, viruses were more often detected in young children, while bacteria and protozoa were more prevalent in youngsters and adults. *C. difficile* was most prevalent in the elderly population (median age 67). Sapovirus differed from the other viruses, as it was only rarely detected beyond 10 years of age (Fig. 2).

**Fig. 3:**
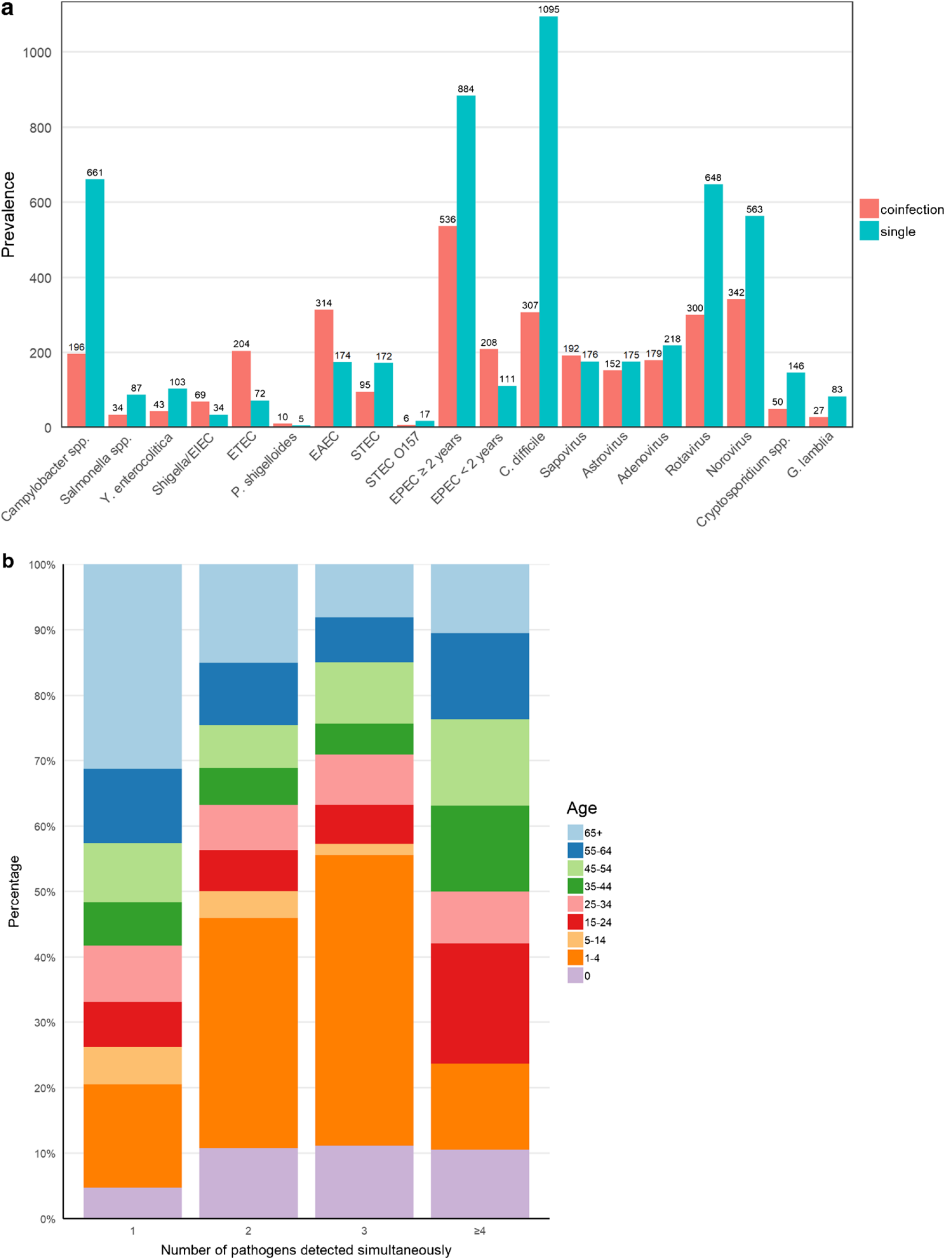
**A** Distribution of single versus coinfections by an enteric pathogen. Pathogens detected < 10 times are not presented. **B** Number of pathogens detected simultaneously by age group

All pathogens were detected as both single and coinfections. Infections with sapovirus, astrovirus, and adenovirus were almost equally distributed between single and coinfections. In contrast, norovirus and rotavirus were detected more often as single infections. *Clostridioides difficile* was the pathogen most frequently detected as a single infection (Fig. 3A). Infections with multiple pathogens, especially with two or three pathogens, were more often detected in young children at age 1–4 years, whereas patients over 65 years of age more often had single infections compared to the other age groups. Coinfections with at least four pathogens were more evenly distributed among different age groups (Fig. 3B).

**Fig. 4:**
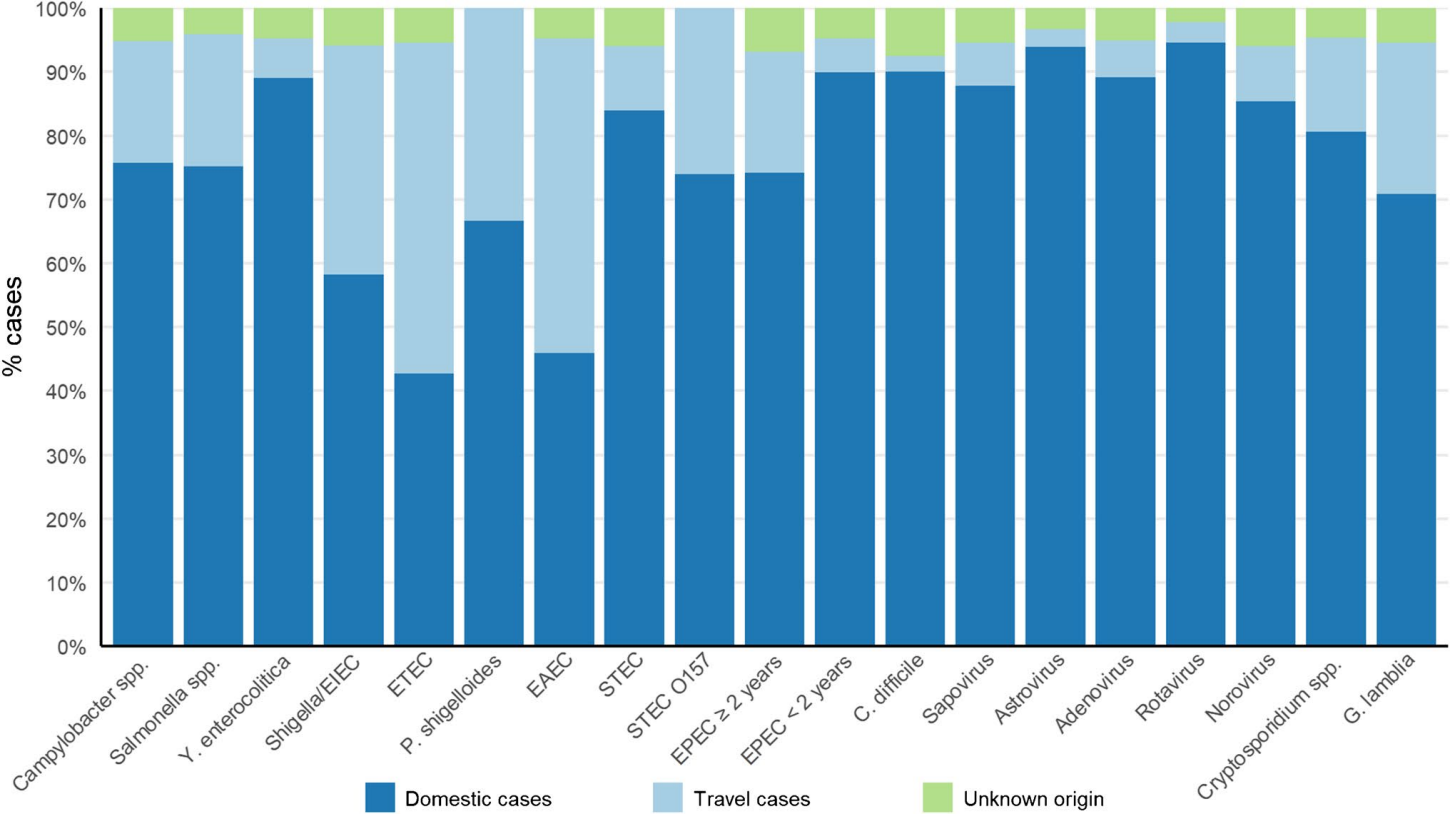
Origin of infections by travel. Pathogens detected < 10 times are not presented

ETEC, *Shigella*/EIEC, EAEC, and *P. shigelloides* were the four bacteria that were most related to travel activity, and coinfections were frequent (Figs. 3A and 4). Of viral cases, 85–90% were domestically acquired. Surprisingly, the majority of protozoan infections were also domestically acquired. No cases of *Entamoeba histolytica* were detected in this study.

**Fig. 5:**
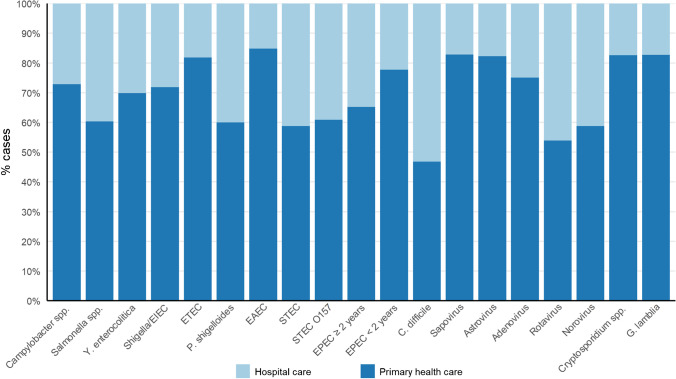
Origin of infections by the healthcare system. Pathogens detected < 10 times are not presented

Origin of infection, i.e., either from patients in the primary healthcare system or hospitalized patients, likely reflects different epidemiology and severity. All pathogens were most often isolated from patients in the primary healthcare system with *C. difficile* as an exception with 53% of cases related to hospital care. Rotavirus and norovirus were detected remarkably more often among hospitalized patients compared to sapovirus and adenovirus (Fig. 5).

**Fig. 6:**
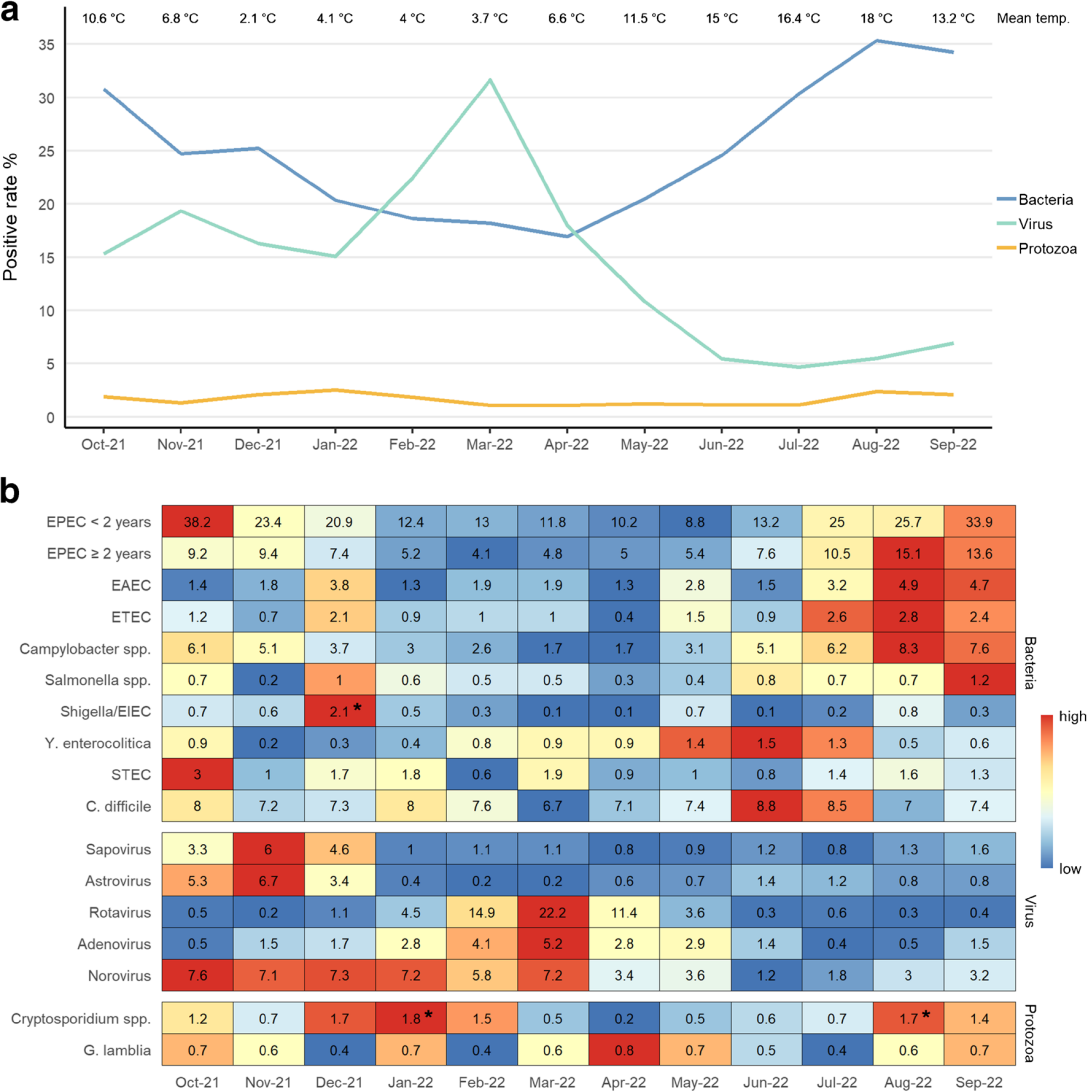
**A** Enteric pathogen group by season. The mean daily temperature per month in Denmark is presented for comparison. In Denmark, placed in the Northern Hemisphere, the annual seasons are winter (December–February), spring (March–May), summer (June–August), and fall (September–October) [9]. **B** Enteric pathogens by month. The colors are based on normalized values for each pathogen and indicate increasing frequency by warmer colors. Numbers in boxes are positive rate (%) of the detected pathogen by the number of tested fecal samples. Rarely detected pathogens (< 25 detections) are not presented. * denotes part of identified and investigated general outbreaks [10, 11]

The proportion of gastrointestinal infections caused by the different groups of pathogens (i.e., bacteria, viruses, and protozoa) varied throughout the year (Fig. 6A). The proportion of viral infections peaked in March (mean ambient temperature 3.7 °C) with a positive rate of 31.6%, and the proportion of bacterial infections peaked in August (mean ambient temperature 18 °C) with a positive rate of 35.3%. The proportion of protozoan infections seemed to be stable throughout the year. Bacteria, especially EPEC, EAEC, ETEC, *Campylobacter* spp., and *Salmonella* spp., had distinct seasonal preferences with the highest number of infections in the warmer summer months (July–September). *Y. enterocolitica* seemed to have a seasonal optimum during the early summer (May–July). The variation of *Shigella*/EIEC, STEC, and *C. difficile* during the year is limited and does not seem to be reliant on temperature. All of the viral pathogens showed distinct seasonal variation. Sapovirus and astrovirus dominated from October to December, whereas rotavirus and adenovirus became dominant from February to April. Norovirus had a high constant prevalence from October to March and was the most dominant virus in the winter half-year (Fig. 6B).

**Fig. 7:**
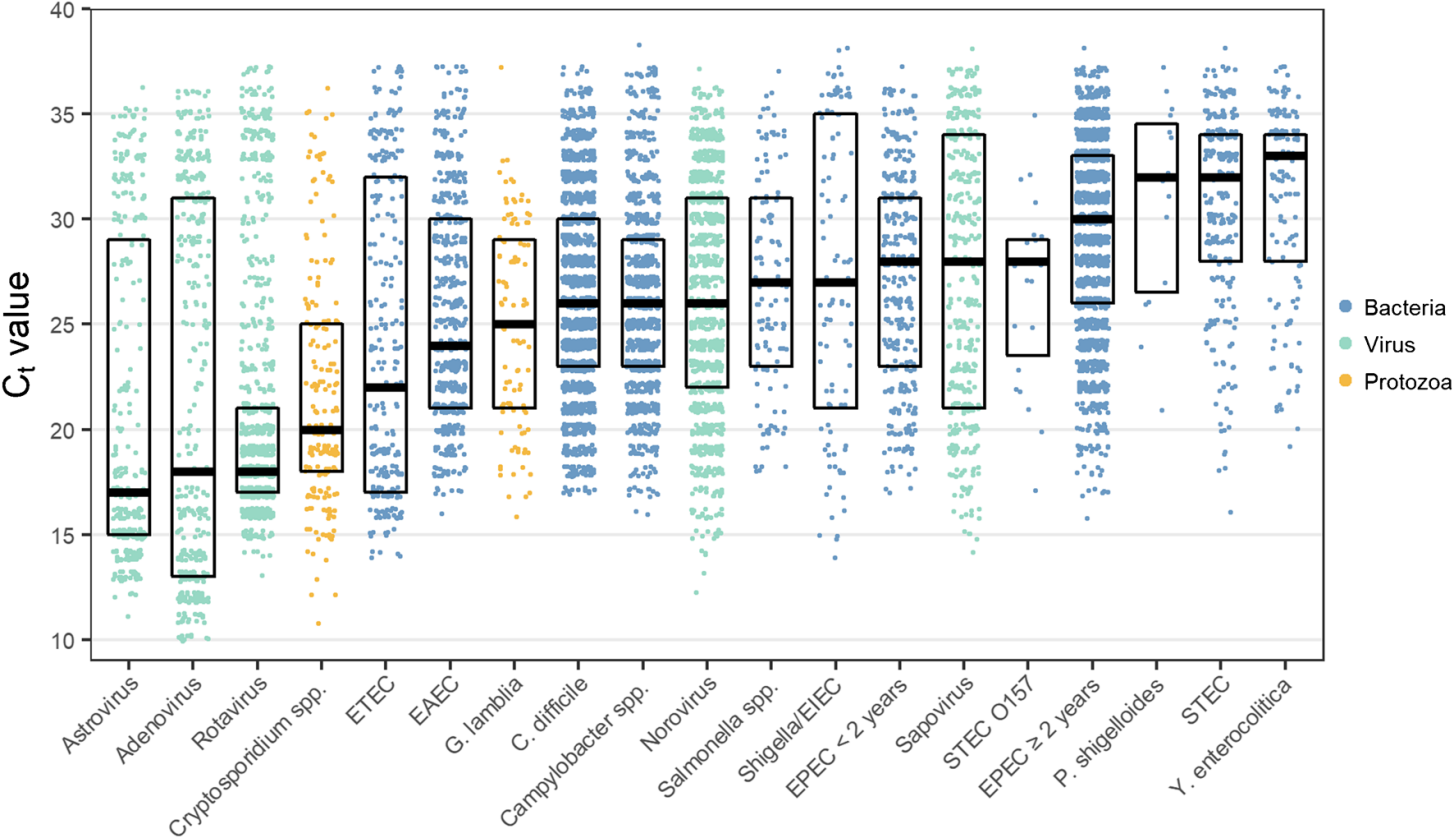
The median and quartiles of PCR *C*_*t*_ values. Pathogens detected < 10 times are not presented

The distribution of *C*_*t*_ values varied significantly between the gastrointestinal pathogens (Fig. 7). The *C*_*t*_ values may be used as indicators for the presence of high or low amounts of DNA/RNA from pathogens in the sample. Astrovirus, adenovirus, and rotavirus had the lowest *C*_*t*_ values with median values of 17, 18, and 18, respectively. In contrast, *Y. enterocolitica* and STEC showed the overall highest *C*_*t*_ values with median values of 33 and 32, respectively. The variation of *C*_*t*_ values within each individual pathogen differed, where rotavirus had a low level of variation, while adenovirus and ETEC had more fluctuating values. Most of the pathogens were in the intermediate group with *C*_*t*_ values of 25–30 (Fig. 7).

## Discussion

Syndromic panel testing to detect infectious microbial pathogens for clinical diagnosis has revolutionized the field of clinical microbiology. We believe this is the first study to perform syndromic testing for a full year, on all diarrheal fecal samples regardless of age, travel history, and clinical indication. We included all diarrheal samples from both in- and outpatients in Region Zealand, Denmark.

We identified a distinct seasonality and age-specific pattern of infections with a high number of viral infections, including coinfections, in children less than 5 years of age in the cold season and a higher number of bacterial infections in adults in the warmer late summer/early fall. These findings were, in part, expected from other European surveillance data [12]. However, there were striking differences within the viral and bacterial groups. The seasonality of astrovirus and sapovirus showed the highest number of infections in the winter, whereas adenovirus and in particular rotavirus peaked in late winter/early spring, and these differences likely reflect differences in optimal conditions including daily ambient temperature for these infections. In March, 22% of the samples were positive for rotavirus. A substantial number of viral infections occurred as coinfections, mostly with other viruses but also with bacteria such as EPEC. The high number of coinfections likely reflects extended excretion time in non-immune children attending daycare, where viral transmission of different viruses and outbreaks frequently occurs [13]. The number of coinfections in the present study is higher than in two distinct Italian and French pediatric studies where coinfections were only detected in 8.3% and 3.3% of virus-positive patients, respectively [14, 15]. In these studies, viruses were mostly detected by EIA and ELISA and some of the differences may be explained by an increased diagnostic sensitivity of the qPCR methods like QIAstat-Dx® Gastrointestinal Panel and FilmArray Gastrointestinal Panel [5]. Sapovirus had a unique age-specific profile with a modest number of infections occurring beyond 10 years of age. Norovirus had a completely different epidemiology with a long winter season, fewer coinfections, and also many cases in adults, where family and small general outbreaks including foodborne outbreaks are well known to occur [16].

The median age of classic food- and waterborne bacterial infections was 35 to 50 years of age with *Shigella*/EIEC somewhat higher (55 years of age). In December 2021, we found five patients with EIEC, which turned out to be index patients in a large national outbreak associated with imported spring onions from Egypt used in ready-to-eat salads [10]. EIEC was subsequently cultured from the majority of PCR-positive fecal samples and typed as part of the outbreak investigations. The domestic outbreak illustrates that unexpected enteric pathogens from ingestion of imported fresh vegetables, fruit, and other food may challenge the evaluation of the PCR, if confirmation by culture or additional tests are negative/not possible. Antimicrobial treatment prior to sampling and liable pathogens like *Shigella* and *Campylobacter* spp. may die before culture and may result in a false negative culture.

STEC including the STEC O157 subtype had the widest 75*th* percentile age profile and was the only bacterial pathogen with a 75*th* percentile age profile including children. This may reflect age-specific exposures to recognized risk factors for STEC (consumption of beef, raw/undercooked meat or cured meat/cold cuts, and contaminated fruits and vegetables) in children and adolescents, but also less immunity to STEC in children [17–19].

EPEC is a principal bacterial cause of diarrheal diseases and outbreaks among infants, in developing countries. It may be associated with severe outcomes including death [20–22]. EPEC’s virulence genes including the intimin gene *eae* are encoded on the chromosomal locus of enterocyte effacement (LEE) pathogenicity island. The QIAstat-Dx® Gastrointestinal Panel detects the *eae* gene and reports positive samples as EPEC positive. In developed countries, such as Denmark, the clinical importance of EPEC in older children and adults is uncertain [23]. For this reason, we analyzed EPEC data < 2 years and ≥ 2 years of age. In patients ≥ 2 years of age, EPEC was the most commonly detected gastrointestinal pathogen with 1421 cases and with a seasonality very different from EPEC < 2 years of age. In addition, the infection was infrequent in older children and adolescents, and the mean age was 47 years of age. This is in line with the findings by Spina et al. using the FilmArray GI Panel [20]. The pathogenic potential of this *E. coli* pathotype in children above 2 years of age and adults remains to be determined and may call for caution to report this pathotype to clinicians, as it may mislead clinicians and delay the diagnosis of other non-infectious diarrheal causes.

*C. difficile* was the most frequently detected pathogen and with the expected highest median age, which is in concordance with earlier studies. *C. difficile* infection (CDI) is a result of prior or current antimicrobial treatment, and some of the detected infections may therefore represent simple carrier states rather than true CDI. For detecting patients at increased risk for clinically significant CDI, some authorities recommend a nucleic acid amplification test (NAAT) like the QIAstat-Dx® Gastrointestinal Panel to be part of a two-step test algorithm [24]. As clinical information was not available, the discussion of the need for a multistep approach is outside the scope of the present study. Because of the high prevalence of asymptomatic carriage of toxigenic *C. difficile* in infants and toddlers, *C. difficile* should not be routinely reported in children less than 2 years of age, or other infectious/non-infectious causes must be excluded [24].

The study uncovered an unexpected high number of *Cryptosporidium* spp. infections including several possible outbreaks. Prior to analysis with the QIAstat-Dx® Gastrointestinal Panel, *Cryptosporidium* spp. was largely undetected in our region, partly because samples rarely were tested for the protozoa and because traditional diagnostic methods have low or moderate sensitivity [25]. In Denmark, it is assumed that transmission occurs through contact with livestock, especially cattle, or through contaminated food, fluids, and recreational water activities [26]. However, Denmark has no national surveillance of cryptosporidiosis, and as an emerging pathogen in Denmark with a likely significant public health impact, an investigation of sources of infections, routes of transmission, and disease burden of infections is urgently needed.

A feature of the QIAstat-Dx® Gastrointestinal Panel is the availability of *C*_*t*_ values and amplification curves for each target detected. In our study, *C*_*t*_ values varied extensively among the pathogens, with the lowest values for astrovirus, adenovirus, and rotavirus. Brown et al. observed that in the pediatric cohort in a UK hospital, norovirus presented significantly lower *C*_*t*_ values than the other viral pathogens [27]. The discrepancy might be partly due to different qPCR methods/platforms used and partly due to the difference in patient groups. Since qPCR analysis can detect the presence of a small amount of pathogen DNA/RNA, a possibility for quantification/semiquantification would be desirable for judging the clinical relevance of the detected pathogen. *C*_*t*_ values could potentially be used as an indicator for the pathogen amount in the sample. Earlier studies observed lower median *C*_*t*_ values in symptomatic patients compared with patients with asymptomatic carriage or colonization [8]. However, one should be cautious to use *C*_*t*_ values as a semiquantitative tool for pathogens. This is due to *C*_*t*_ values depending on pathogen load, preanalytic factors like prior antimicrobial treatment, sampling, transport time, storage of specimens, and analytical factors including DNA/RNA extraction, the primer design, and the effectivity of the detection of amplified products [6].

There are limitations associated with this study. First, qPCR analysis is extremely sensitive, allowing for the detection of very small amounts of pathogens, which may or may not represent biologically or clinically relevant infections. There is a lack of knowledge on the length of excretion of detectable DNA/RNA of enteric pathogens after diarrheal episodes and in different age and patient groups. In addition, some pathogens including EPEC ≥ 2 years old and EAEC asymptomatic carriage in epidemiological studies indicate inconsistent relationships between marker gene presence and diarrheal symptoms. The laboratory may not report pathogens with incomplete established diarrhoeagenic potential or qualify test reports with added written clinical guidance. In addition, clinicians need to provide an educated judgment on the clinical relevance of the results and/or rely on baseline data. Furthermore, clinicians may be encouraged to qualify test requests, for example, for community-acquired diarrhea only to request a test if the duration of the diarrhea has been > 7 days, diarrhea with warning signs/risk factors for severe disease, or suspicion of an outbreak.

No *E. histolytica* was found in this study, but the Parasitic Gastroenteritis EQA programs from QCMD has in our hands demonstrated QIAstat-Dx’s ability to detect *E. histolytica* in the EQA programs from 2021 and 2022.

For economic reasons, we did not perform repeated testing (within 7 days) regardless if the first sample was positive or negative. However, it cannot be excluded that some patients were additionally tested with QIAstat-Dx® Gastrointestinal Panel after 7 days for the same diarrhoeal episode.

During the COVID-19 epidemic, a general decrease in the number of diarrheal infections was observed in Denmark [28]. This study was performed after the end of the Danish travel restrictions implemented during the COVID-19 epidemic. However, this was not the case for all typical Danish travel destinations, and an impact of persistent COVID-19 travel restrictions may have had an impact on the total number of detected infections and on the number of travel-related infections in the present study. A drawback of the QIAstat-Dx® Gastrointestinal Panel is the cost per sample. However, the test expenditure may be compensated by savings in clinical wards by decreasing the time to diagnosis, contact precautions, unnecessary diagnostic examinations, and shorter lengths of stay at the hospital.

For patients care and for local, regional, and national surveillance purposes, selected enteric pathogens should subsequently be further characterized (culture, antibiogram testing, and/or molecular typing, including by whole-genome sequencing (WGS), but this is out of scope for the present study.

## Conclusions

Routine use of the QIAstat-Dx® Gastrointestinal Panel identified a large range of gastrointestinal pathogens, including earlier underdetected pathogens like *Cryptosporidium* spp., in 37% of all the fecal samples during a 1-year period in Region Zealand, Denmark. Distinct seasonality and age-specific patterns of infections were observed for several pathogens, especially viral pathogens. All pathogens were detected in both single and coinfections, though with varied distribution. The QIAstat-Dx® Gastrointestinal Panel with its ease-to-use and short turn-around time is a useful tool for diagnostic testing of gastrointestinal infections, both for in- and outpatients.

## References

[1] Salam MA, Advisor S, Lindberg G, Dite P, Khalif I, Salazar-Lindo E (2013). Acute diarrhoea in adults and children - a global perspective. J Clin Gastroenterol.

[2] Vos T, Allen C, Arora M, Barber RM, Bhutta ZA, Brown A (2016). Global, regional, and national incidence, prevalence, and years lived with disability for 310 diseases and injuries, 1990–2015: a systematic analysis for the Global Burden of Disease Study 2015. Lancet.

[3] Boers SA, Peters CJA, Wessels E, Melchers WJG, Claas ECJ (2020). Performance of the QIAstat-Dx gastrointestinal panel for diagnosing infectious gastroenteritis. J Clin Microbiol.

[4] Zhang H, Morrison S, Tang YW (2015). Multiplex polymerase chain reaction tests for detection of pathogens associated with gastroenteritis. Clin Lab Med.

[5] Calderaro A, Martinelli M, Buttrini M, Montecchini S, Covan S, Rossi S (2018). Contribution of the FilmArray® Gastrointestinal Panel in the laboratory diagnosis of gastroenteritis in a cohort of children: a two-year prospective study. Int J Med Microbiol.

[6] Castany-Feixas M, Simo S, Garcia-Garcia S, Fernandez De Sevilla M, Launes C, Kalkgruber M (2021). Rapid molecular syndromic testing for aetiological diagnosis of gastrointestinal infections and targeted antimicrobial prescription: experience from a reference paediatric hospital in Spain. Eur J Clin Microbiol Infect Dis.

[7] Engberg J, Vejrum LK, Madsen TV, Nielsen XC (2021). Verification of analytical bacterial spectrum of QIAstat-Dx®GI V2 and Novodiag® Bacterial GE+ V2-0 diagnostic panels. J Antimicrob Chemother.

[8] Bonacorsi S, Visseaux B, Bouzid D, Pareja J, Rao SN, Manissero D (2021). Systematic review on the correlation of quantitative PCR cycle threshold values of gastrointestinal pathogens with patient clinical presentation and outcomes. Front Med.

[9] The Danish Meterological Institute (DMI) [Weather archive] (2022) (available at https://www.dmi.dk/vejrarkiv). 2022-11-14

[10] Torpdahl M, White ED, Schjørring S, Søby M, Engberg J, Engsbro AL (2023). Imported spring onions related to the first outbreak with enteroinvasive Escherichia coli in Denmark, November to December 2021. Euro Surveill.

[11] Stensvold C, Hartmeyer G, Schouw C, Aftab H, Engberg J, Nielsen H (2022). Investigation into Cryptosporidium clusters identified in neighboring regions in Denmark in December 2021- January 2022 – separate or linked? ICOPA 2022.

[12] European Food Safety Authority (2021). European Centre for Disease Prevention and Control, The European Union One Health 2020 Zoonoses Report.

[13] Hebbelstrup Jensen B, Jokelainen P, Nielsen ACY, Franck KT, Rejkjær Holm D (2019). Children attending day care centers are a year-round reservoir of gastrointestinal viruses. Sci Rep.

[14] De Grazia S, Bonura F, Bonura C, Mangiaracina L, Filizzolo C, Martella V (2020). Assessing the burden of viral co-infections in acute gastroenteritis in children: an eleven-year-long investigation. J Clin Virol.

[15] Tran A, Talmud D, Lejeune B, Jovenin N, Renois F, Payan C (2010). Prevalence of rotavirus, adenovirus, norovirus, and astrovirus infections and coinfections among hospitalized children in Northern France. J Clin Microbiol.

[16] Espenhain L, Berg TC, Bentele H, Nygård K, Kacelnik O (2019). Epidemiology and impact of norovirus outbreaks in Norwegian healthcare institutions, 2005–2018. J Hosp Infect.

[17] Tack DM, Kisselburgh HM, Richardson LC, Geissler A, Griffin PM, Payne DC (2021). Shiga toxin-producing Escherichia coli outbreaks in the United States, 2010–2017. Microorganisms.

[18] Statens Serum Institut (2019) STEC - report on disease occurrence 2014-2018. https://www.ssi.dk. Accessed 11 October 2022

[19] Beutin L, Fach P (2014) Detection of Shiga toxin-producing Escherichia coli from nonhuman sources and strain typing. Microbiol Spectr 2(3). 10.1128/microbiolspec.EHEC-0001-2013 10.1128/microbiolspec.EHEC-0001-2013 26103970

[20] Spina A, Kerr KG, Cormican M, Barbut F, Eigentler A, Zerva L (2015). Spectrum of enteropathogens detected by the FilmArray GI Panel in a multicentre study of community-acquired gastroenteritis. Clin Microbiol Infect.

[21] Sakkejha H, Byrne L, Lawson AJ, Jenkins C (2013). An update on the microbiology and epidemiology of enteropathogenic Escherichia coli in England 2010-2012. J Med Microbiol.

[22] Kotloff KL, Nataro JP, Blackwelder WC, Nasrin D, Farag TH, Panchalingam S (2013). Burden and aetiology of diarrhoeal disease in infants and young children in developing countries (the Global Enteric Multicenter Study, GEMS): a prospective, case-control study. Lancet.

[23] Berdal JE, Follin-Arbelet B, Bjørnholt JV (2019). Experiences from multiplex PCR diagnostics of faeces in hospitalised patients: clinical significance of enteropathogenic Escherichia coli (EPEC) and culture negative campylobacter. BMC Infect Dis.

[24] McDonald LC, Gerding DN, Johnson S, Bakken JS, Carroll KC, Coffin SE (2018). Clinical practice guidelines for Clostridium difficile infection in adults and children: 2017 update by the Infectious Diseases Society of America (IDSA) and Society for Healthcare Epidemiology of America (SHEA). Clin Infect Dis.

[25] Checkley W, White AC, Jaganath D, Arrowood MJ, Chalmers RM, Chen XM (2015). A review of the global burden, novel diagnostics, therapeutics, and vaccine targets for Cryptosporidium. Lancet Infect Dis.

[26] Stensvold CR, Ethelberg S, Hansen L, Sahar S, Voldstedlund M, Kemp M (2015). Cryptosporidium infections in Denmark, 2010-2014. Dan Med J.

[27] Brown JR, Shah D, Breuer J (2016). Viral gastrointestinal infections and norovirus genotypes in a paediatric UK hospital, 2014–2015. J Clin Virol.

[28] Anonymous (2021) Annual report on zoonoses in Denmark 2020. (https://www.food.dtu.dk). Accessed 20 January 2023

